# Reported impact of creativity in the Wakakosha (‘You’re Worth It’) internal stigma intervention for young people living with HIV in Harare, Zimbabwe

**DOI:** 10.1371/journal.pgph.0003909

**Published:** 2024-11-05

**Authors:** Lauren Heniff, Nadine Ferris France, Webster Mavhu, Mohannad Ramadan, Owen Nyamwanza, Nicola Willis, Eimear Crehan, Moud Chinembiri, Deirdre Ni Cheallaigh, Ann Nolan, Elaine Byrne

**Affiliations:** 1 Trinity College Dublin, Dublin, Ireland; 2 Beyond Stigma, Dublin, Ireland; 3 Department of Public Health and Epidemiology, University College Cork, Cork, Ireland; 4 Centre for Sexual Health and HIV/AIDS Research (CeSHHAR), Harare, Zimbabwe; 5 Department of International Public Health, Liverpool School of Tropical Medicine, Liverpool, United Kingdom; 6 Hashemite University, Az Zarqa, Jordan; 7 Zvandiri, Harare, Zimbabwe; 8 Speak Up Sing Out, Meath, Ireland; 9 Beyond Stigma, Harare, Zimbabwe; 10 Centre for Positive Health Sciences, Royal College of Surgeons in Ireland, Dublin, Ireland; PLOS: Public Library of Science, UNITED STATES OF AMERICA

## Abstract

Internal stigma (negative judgements towards oneself) continues to be a barrier to HIV treatment, management and care, and has global public health consequences. People living with HIV (PLHIV) who report internal stigma are less likely to seek care, adhere to treatment and can experience increased depression and lower quality of life. The *Wakakosha* (‘You’re Worth It’) programme sought to reduce internal stigma among young PLHIV using inquiry-based stress reduction (IBSR), a cognitive and awareness-based methodology. This sub-study focused on creativity, exploring how it promotes young PLHIV’s well-being. We analysed individual interviews (n = 14), focus groups (n = 3), poems (n = 5), songs (n = 2) and self-compassion letters (n = 38) and 23 activity journals from November 2021 to March 2022, utilising inductive thematic analysis to identify themes across the data. Creativity saturated the *Wakakosha* intervention through modalities such as drawing, colouring, body mapping, music and letter writing. These engaged participants and gave them a space in which to shift their internal stigmatising beliefs, and helped them in multiple ways including: facilitating emotional regulation, self-acceptance, self-compassion, self-worth and body positivity. Creativity also enhanced participants’ self-image and allowed them to see their own abilities. Music improved the therapeutic environment by helping participants focus, emote, retain messages and connect within the group setting. Letters to self/others allowed participants to let go of stigma towards themselves and the circumstances of their infection. Tools such as drawing and body mapping assisted participants with emotional recognition and expression. This sub-study provided insight into the potential of creativity, when integrated into an IBSR intervention, in counteracting internal stigma. Ongoing replication is needed to continue to evolve best practices for internal stigma interventions. Future work should use more structured and specific interviews with participants regarding their creative processes.

## Introduction

More than four decades into the global HIV pandemic, stigma remains a major barrier to HIV treatment, management and care despite the great strides in improving the quality of life and longevity of people living with HIV (PLHIV) [1–4]. Stigma emerges from a dynamic social process that is enacted and perpetuated through individuals, communities and society [5]. PLHIV experience public stigma—negative beliefs held at the societal level and the abusive actions they can often generate [6,7]. Relatedly, they experience interpersonal discrimination from their immediate circle—including family members, peers and health personnel [8–12].

PLHIV also experience internal stigma. Alternatively known as self, personal or internalised stigma, internal stigma is experiencing negative judgements towards oneself, resulting in feelings of shame, worthlessness, self-blame and emotional distress [13]. A systematic review found that HIV-related stigma, including internal stigma, undermined HIV medication adherence by compromising general psychological processes (e.g., adaptive coping and social support) [14]. In the same review, 70% of the studies reported an association between HIV stigma and medication non-adherence [14]. Internal stigma is experienced by PLHIV to a far greater extent than stigma received from the broader community [15,16].

A previous study on HIV in Zimbabwe indicated that 73% of adolescents living with HIV had experienced stigma that affected their adherence to medication [17]. A separate qualitative study of Zimbabwean adolescents and young adults living with HIV identified relationships, isolation and disclosure as the main experiences that were associated with internal stigmatisation [18]. The same study also suggested that young PLHIV were particularly likely to internally stigmatise because of the condition [18].

The creative arts have proven to be an effective medium of information dissemination and stakeholder engagement in promoting health and well-being [19]. Among individuals with a wide spectrum of conditions including cancer, leukaemia, poor mental health and HIV, health messages have been conveyed in art forms including music, theatre, art and dance, among others, with remarkable outcomes for healing and well-being [20–24]. Notably, for children, adolescents and young adults, including those living with HIV, creative arts present safe havens which enable them to face their fears and anxieties [22–25]. Creativity allows them to express and manage their emotions, while also serving as a positive distraction from physical, psychosocial, mental and other problems associated with any condition [26–28].

A scoping review commissioned by the World Health Organisation explored over 3,000 studies at the intersection of the arts and improvement of health and well-being [29]. The review concluded that creative expression could promote holistic well-being through connection and expression, and can be facilitated in a therapeutic context [29]. The creative expression that takes place in arts participation can cultivate problem-solving, creative thinking and promote the reconstruction of beliefs [29]. Although there is a wealth of research on creativity-oriented interventions, very few focus on creativity and internal stigma.

Here, we describe findings from an initiative that explored participants’ perspectives on the impact of creativity embedded within *Wakakosha* (‘You’re Worth It’), an intervention to support young PLHIV to move from positions of internal stigma (negative) to self-worth (positive) [30]. We aimed to broaden the knowledge base on creativity in the therapeutic context and provide insight into how internal stigma interventions might be optimised.

## Materials and methods

### Study design: Intervention overview

The *Wakakosha* (‘You Are Worth It’) intervention has been previously described in detail [30]. In brief, we sought to adapt and optimise an innovation we previously piloted among adults living with HIV in Zimbabwe–Inquiry-Based Stress Reduction (IBSR): The Work of Byron Katie [13]. IBSR is a cognitive and awareness-based approach that provides a unique way of identifying and questioning thoughts that cause shame, anger, fear and violence. The *Wakakosha* intervention aimed to equip adolescents and young PLHIV (AYPLHIV, aged 18–24 years) in the use of IBSR/self-inquiry to manage stressful beliefs, reduce internal stigma and grow their sense of self-worth and well-being. The intervention consisted of 16x3-hour sessions delivered weekly or once as a 10-day workshop, and included individual and pair work [30].

Each session contained a mix of theory, meditation, group and individual experiences of IBSR, reflection and sharing of insights, music, singing and songs carefully matched to a session’s themes and content to enhance learning and healing. Supporting the intervention was a 156-page activity journal that contained worksheets, exercises and homework. Techniques such as journaling, box-breathing, podcasts, letter writing, poetry and drawing were used throughout [30]. Towards the end, participants produced a song and recorded a music video which showed, demonstrated and expressed their experiences of the programme and what happens when internal stigma is addressed. They also produced a podcast series through which they shared their own letters about the body and forgiveness (available: https://www.beyondstigma.org/videos). Additionally, they produced a poetry book (available: https://www.beyondstigma.org/poetry)

### Data collection and analysis

In this paper, we describe secondary analysis of data we previously collected and reported as part of the *Wakakosha* programme evaluation [30]. As part of that evaluation, firstly, in November 2021, we held a focus group discussion (FGD) with 12/30 Community Adolescent Treatment Supporters (CATS, peer supporters) who were the first to receive the *Wakakosha* intervention. We also held in-depth interviews (IDIs) with another 12 CATS between November 2021 and February 2022. Additionally, we held a FGD with 12/15 CATS recently trained as peer *Wakakosha* intervention coaches in March 2022. Further, we held two separate FGDs with 24/30 CATS recently trained by peer coaches in November 2022. In addition, we conducted key informant interviews (KIIs) with 2/4 adult coaches who had facilitated the intervention sessions (Table 1). We examined the same data, but looking specifically at creativity and its reported impact, effect and influence. We additionally used a short poem anthology (n = 5), songs (n = 2), activity journals (n = 23) and letters to self/others (n = 38) to augment the interviews and FGDs.

**Table 1:** Summary of qualitative data collection.

Date	Participant Category	Method	Number
Nov 2021	First set of CATS	FGDx1	12 (n = 6 female)
Nov 2021- Feb 2022	First set of CATS	IDIx12	12 (n = 6 female)
March 2022	Peer Coaches	FGDx1	12 (n = 6 female)
November 2022	Second set of CATS (trained by peer Coaches)	FGDx2	24 (n = 12 female)
November 2022	Adult local Coaches	KIIx2	02 (n = 2 female)

We analysed the data thematically [31], with interviews and FGD transcripts being imported into NVivo 14 (QSR International, Melbourne, Australia). We then carried out a phased process to systematically process the data, starting with data familiarisation, including reading through the transcripts. Secondly, we created initial codes and categories to capture common topics in the data and categories relevant to the research questions. LH, EB and NFF discussed the initial codes and agreed on their suitability. Thirdly, we generated themes and sub-themes from the initial codes. Fourthly, LH reviewed the themes/sub-themes in consultation with an advisor (MR) who gave his input on their validity. We subsequently defined and named the themes/sub-themes and synthesised findings.

### Ethical considerations

The Zimbabwe national ethics committee, Medical Research Council of Zimbabwe approved this study (#2604), and all participants provided written informed consent prior to their participation. We de-identified interviews and FGD transcripts before analysis and generated codes to identify different participants. We stored data on a secure, encrypted cloud-storage which was only accessed by authorised team members.

#### Inclusivity in global research

Additional information regarding the ethical, cultural and scientific considerations specific to inclusivity in global research is included in S1 Checklist.

## Results

*Wakakosha* creative activities were helpful to participants in multiple ways, including facilitating emotional regulation, self-acceptance, self-compassion, self-worth and body positivity. Both the creative activities and their reported transformative aspects are discussed in detail below.

### Emotional regulation

Creative activities that had an emotion-regulating impact on *Wakakosha* participants included compositions (themed pre-recorded music/songs, singing, poetry) and virtual representations (drawing, colouring, Zen doodling, body mapping).

#### Music/Singing

Singing was a regular feature throughout the 16-session intervention and participants contended that singing as a group had a bonding effect as it drew them closer to each other. Not only was music unifying but it was also therapeutic, with participants acknowledging its soothing effect. The background music during sessions was also noted to significantly help participants focus and connect with their emotions. One participant remarked that, *‘For instance*, *when we were doing tasks that required concentration*, *they (facilitators) would play some soft music in the background*, *and it would make me focus’* (Female 22yrs, FGD 2). Another participant highlighted the emotive power of the intervention songs as well as their ability to prompt deep meditation, stating that, *‘It (music) helped me meditate*, *I would sit and take in the message of the song before starting to write*…*’* (Female 20yrs, FGD 1).

Speaking on the emotion-regulating influence of music, one participant noted the deeply soothing effects of the lyrical content of the songs they sang, stating that, *“They (the songs) were helpful because when you sang*, *the lyrics would really go into your heart*, *and you were assisted emotionally and also with stressful thoughts”* (Male, 22yrs FGD1). Not only was music effective in emotion regulation, but it also helped participants with stress management. ‘*And also*, *I learnt to manage stress*, *what to do when I am stressed*. *I now listen to music*, *and I feel much better after listening to music’* (Male 22yrs, FGD1).

There was a degree of relatability between the intervention songs and participants’ lived experiences. One participant commented that, *‘The song titled “Lord Don’t Move That Mountain” taught me a lot*. *It is just a song*…*but I used to ask myself why I am HIV positive*. *It made me realise that*, *this is a mountain that has been placed on me by God and He cannot move it*. *He can only give me the strength to climb the mountain and accept my [HIV] status’* (Male 19yrs, FGD 3). In the case of the main *Wakakosha* song, participants were particularly emboldened by the lyrics which encouraged them to be proud of who they were as well as to reinforce the self-worth message as captured below:

*‘I can feel love and be proud of my body, it’s my friend*

It was perfectly made, made from God, we won’t part till the end

People with HIV can do anything, we can be free

*Positive or negative*, *we are the same you and me’*

Another verse also written by the young people states:

*‘Self-stigma stops me doing what I wanna do*

I have to take a deep breath; I know I can get through

I am who I am and I am learning how to love

*There is so much stigma*, *but I will rise above’*

Creativity was directly tied to the curriculum. For example, the lyrics above were collected after IBSR sessions where participants worked on stressful thoughts about their bodies, also how they felt judged by society because of their HIV status, and how they stigmatised themselves.

One participant stated that they drew inspiration from the lyrics to the song titled *“Every little cell”*:

*‘Every little cell in my body is happy,*

Every little cell in my body is well.

I’m so glad every little cell

*In my body is happy and well…’*

The song conveyed a message of body positivity, which most participants notably struggled with prior to the intervention, prompting them to self-stigmatise.

#### Poetry

*Wakakosha* participants also used poetry ‘*…to express what seems inexpressible*, *our hopes*, *our dreams*, *our personal truths*, *our moments of transformation our love…’* (Foreword, *Wakakosha* Poetry Booklet). Participants composed short poems particularly expressing their feelings about their internal stigma experiences as well as to express their gratitude to their bodies for enduring the stress. Contributors to the anthology acknowledged the transformative and healing power of poetry, noting that, ‘*When I wrote my poem*, *I was heartbroken*, *did not have confidence (and) looked down upon myself… After finishing the poem*, *I was happy because I felt lighter*, *like a heavy weight was removed’* (Female poet 1 testimony, *Wakakosha* Poetry Booklet).

A participant reported how they *‘…experienced some supernatural relief inside my heart as I was writing’* (Male poet 1 testimony, *Wakakosha* Poetry Booklet). Yet another participant noted that poetry writing gave them the opportunity to recall and decant all the bad memories of their upbringing, leaving them *‘…relieved and stress free’* (Female poet 1 testimony, *Wakakosha* Poetry Booklet). Similarly, one participant characterised the poetry writing process as ‘*lifting a burden’* in their life (Male poet 2 testimony, *Wakakosha* Poetry Booklet). A key message in the selected poems relates to hope for a better future that participants had despite the many challenges they regularly encountered. The poetry anthology produced was called ‘There is a treasure in me’ (available: https://www.beyondstigma.org/poetry).

#### Drawing and colouring

Drawing and colouring were designed to offer participants an opportunity to relieve distress while also enhancing their capacity to express themselves. Specifically, drawing was infused into the intervention to serve as a ‘distraction’ from the stresses arising from internal stigma. In most interviews, participants showed appreciation of the positive impact that drawing had had on them. One participant acknowledged that the drawing and colouring activities brought the ‘inner child’ in them to the fore, stating that, *‘The drawings were helpful to me*. *I could feel like a kid…when you give a child papers*, *she will become focused on the papers*. *I will not be thinking about anything surrounding me*, *I will be concentrating on that (drawing)’* (Female 23yrs, IDI).

Colouring was also notably crucial in emotion regulation, with participants still using the skill beyond the intervention phase as acknowledged in this assertion. ‘*There were colours that we used to colour our moods*. *We would use a certain colour to colour certain emotions we were feeling*. *As I discovered*, *it was a way of reducing stress*. *Sometimes when you start to write in colours you are now interested in colouring your emotions*. *I noticed that it was a method that helped me*. *Even now I can still do these things when I am stressed’* (Male 22yrs, IDI).

#### Zen doodling

Exercises in Zen doodling (a style of doodling/drawing where one creates intricate designs by completing small areas of patterns), were also integrated into the drawing activity as a stress management strategy, and participants were positively impacted by them, with one participant noting that, *‘We were taught to appreciate your body…or that when you are stressed you can take time to draw doodles so that you become relieved’* (Female 23yrs, IDI).

Viewed as visual representations, drawing and colouring occasionally served as a means of releasing suppressed feelings, particularly when participants associated the act with “sharing” and “de-stressing”. *‘I am not good at sharing*, *but I discovered that when I am using that paper*, *I am actually sharing my problems*. *I am exchanging words with my paper*, *sharing my stressful thoughts for that particular day*. *Then in terms of colours and colouring*, *I have realised that when I am colouring*, *I will be de-stressing’* (Female 22yrs, FGD1).

#### Body mapping

In this activity, participants drew an outline of their body on paper and coloured on this image to identify their emotions and where they “felt” them. This process prompted them to reflect on and visualise their emotions and the resultant body maps assisted them in emotional recognition and expression. An integral part of activity journaling, body mapping at the beginning of sessions served to ignite awareness of the feelings one may be grappling with at that time. Body mapping was also used to signal the degree of distress one may be going through. For instance, one participant indicated that colouring within body mapping using certain colours, as well as targeting certain body parts (the forehead or whole body), signified the depth of the stress he was having. *‘When I was happy*, *I usually used green*. *I would use red when I was in a bad mood*. *Or colour the forehead when I was angry*. *When I was in a really bad mood*, *I would paint the whole body in red and black to show that things were not okay at all’* (Male, 20yrs, IDI).

The importance of colouring alongside body mapping as an emotion-tracing strategy was acknowledged even by facilitators as it helped them diagnose problems affecting the participants.

*‘So, with regards to colouring, I gained that colouring skill. (And) now when I am with adolescents, I can just give them colours and ask them to colour the emotions they are having. Sometimes they might not even know that I am doing this for mental health. But as they are doing it, I can then conclude that, that child is being stressed by this so that I can help her using that skill’* (Facilitator 1, KII).

All the activities described above were included in the Activity Journal each participant received.

### Self-acceptance, self-compassion, forgiveness and self-worth

Through different creative activities, including those aimed at questioning one’s thoughts, participants developed a sense of acceptance of their situation as AYPLHIV and its related challenges. Particularly, through writing letters to self or others, participants were able to develop feelings of acceptance of their situation and adequate coping mechanisms to overcome the associated internal stigma. Letter writing was guided by the structure of: “3 things you did to hurt yourself, how can I make it right, and 3 things I am grateful to myself for”. Below is a letter-to-self by one participant expressing their regret for neglecting their body owing to internal stigma:

*Dear Body*

*My dearest body*, *I’m sorry I hurt you terribly by not eating well and starving you* ***because of my self-stigma***. *I lost control and I could not concentrate on you*. *I deserted you and I cried all my eyes out all the night*. *I did not take my medication well and I spoiled your normal function*. *I am GRATEFUL you managed to fight the pain and control yourself*. *I’m humbled by you being able to focus and strengthen yourself when I was starving you*. *You were so much patient with me going through the rough patch*, *and I’m so honoured by your resilience*. *Keep going*! (‘Dear Me’ Podcast #4).

Participants acknowledged that these letters were a turning point in their lives. They provided them with space to expressly question their thoughts, make personal commitments to treat their bodies better, as well as to vent the anger and resentment towards others. In one letter, the writer sought self-forgiveness and pledged to positively embrace their condition going forward, noting that, *‘Please forgive me [self]*, *I choose to be in love with you today*. *I will no longer be ashamed of you’* (Dear Me Podcast #5).

Similarly, they sought forgiveness from their significant others for transgressing in different ways, while also accepting their situation and living positively with it. Acceptance and forgiveness are evident in the sentiments of one participant who sought forgiveness from their mother for previously blaming her. ‘*I wrote a letter to my mother asking for forgiveness*. *I was angry due to my status*, *saying*, *“I am HIV positive because you were careless”‘* (Male 19yrs, IDI). Through exposure to knowledge and information, participants started to accept that the vertical HIV transmission was not intentional. *‘I would blame her [mother] (prior to the intervention) but I realised that during that time*, *there was no medication to prevent HIV*. *So*, *it was not her fault at all’* (Male 19yrs, IDI). Another male participant described how the *Wakakosha* activities had helped him find closure. *‘So*, *I wrote a letter (as part of intervention activities) to both of them (deceased parents) asking for forgiveness…’* (Male 22 years, IDI). He went on to describe what he did next. *‘One weekend I travelled to the rural areas*, *bought flowers*, *and went to their graves and ‘said’ to them “I am very sorry for not forgiving you all along…” I then placed the flowers on their graves’* (Male 22 years, IDI).

Participants reported being positively impacted by the intervention in their self-image and beliefs about their capabilities. Specifically, they drew inspiration from letter writing and music activities, and they reported experiencing a shift in mental state, which helped them to value themselves more. One participant acceded that music *‘…helped a lot because it left you motivated and (believing) that you are worth it*. *For example*, *the Wakakosha song that we sang*. *I never thought I could be that important*. *When XX led the song*, *I would start meditating that there was a time when I looked down upon myself*. *But when I sang the song*, *I could feel that I was worthy…”* (Male 22yrs, IDI).

The creative activities also ignited a sense of renewed self-worth among participants. Because of internal stigma, participants often looked down upon themselves and had limited confidence. However, with the help of creative activities in *Wakakosha*, their self-worth was significantly uplifted. One participant noted that by writing a letter to the body, they were able to re-affirm their self-worth. *‘I wrote a letter apologising about what I used to do…I would isolate myself*. *I then realised that this was not healthy because I was always stressed…I would sit alone but now I have changed’ (Male 20yrs*, *IDI)*.

Equally, the self-worth message resonated across the *Wakakosha* short poetry anthology. In one poem, the author reaffirmed their renewed sense of self-worth by likening themselves to royalty, declaring, ‘*How amazing it is to be of the royal blood…’* and boldly proclaiming that ‘*Greatness is your portion and part of your life’* (Male poet 3, *Wakakosha* Poetry Booklet).

### Body positivity

Creative activities also promoted body positivity among all participants. This was particularly observed for participants who were previously too self-conscious of their ‘blemished’ body (stunted due to HIV or because of mottled skin texture). Participants’ relationships with their bodies were key mediators in the process of self-stigmatisation in that the body was a place of the self and a place where HIV was housed and experienced. Commenting on internal stigma related to body consciousness, one participant remarked that, *‘I used to be ashamed of my tummy*, *which is slightly bigger*. *I would think that people would laugh at me*, *for instance*, *if I were to dance in front of them*. *I was too conscious of my body because of my status*, *I felt like my status was projected onto my body’* (Male 21yrs, FGD2). Another remarked that, *‘I used to “hate” my body*, *and I would say*, *“I don’t want my body because I am tiny*. *I don’t want my skin because it is too dark” (Female 24yrs*, *FGD1)*.

Through various creative activities, participants assumed an improved outlook of their bodies. Summarising contents of the letter he had written to his body, one participant acknowledged the body’s resilience. *‘I thanked it (body) for enabling me to qualify for a soccer tournament*. *I would compete with the tall ones*, *yet I am slim and short [stunted due to HIV]’* (Male 19yrs, FGD 1). Noteworthy was the body positivity message that the creative activities inculcated in participants, motivating them to reevaluate their attitudes and beliefs towards their bodies and embracing body positive routines. A participant stated that, ‘It was only after *Wakakosha* when I realised that I should be proud of my body because my face has not been reacting to the drugs that I am taking’ (Female 23 years, IDI). More so, a recurring message in the Dear Me Podcasts is that of participants committing to taking better care of their bodies through improved hygiene, eating healthy and quitting alcohol/drugs (see Dear Me Podcast #11, #12, #13).

## Discussion

We explored the impact of various creative activities that were embedded within the *Wakakosha* intervention and found that the activities were impactful in multiple ways, including facilitating emotional regulation, self-acceptance, self-compassion, self-worth and body positivity. This study is one of the few to focus on creativity and internal stigma among young PLHIV in sub-Saharan Africa. Other work has focused on different aspects of creativity and HIV, such as applied sculpture, drawing, song and dance [23,24,32] and art festivals [33] but more in the context of health promotion, awareness around HIV and empowerment. A recent systematic review identified 35 interventions to reduce HIV-related internal stigma [34]. While some interventions contained creative approaches, only a few within the categories of multi-pronged and mindfulness-based approaches used creativity explicitly. Findings from the research reported here will help broaden the knowledge base on creativity in the therapeutic context, and provide insight into how internal stigma interventions, including those targeting HIV internal stigma, might be optimised.

Through different creative activities, particularly letter writing, participants were able to express themselves and find their voices while feeling calm and present. The letter writing activity also afforded participants a chance to accept their situation and to foster a sense of forgiveness to self and those they blamed for their predicament. Self-acceptance and (self-) forgiveness are critical ingredients to reduction of internal stigma, and a pivotal turning point towards positive attitudes for intervention participants [35–37]. Self-forgiveness notably constitutes a natural repair mechanism which cultivates a sense of inner peace for those seeking it [38]. This resonates well with the work of Griffiths which, while not focusing exclusively on HIV-related internal stigma, established that creative activities generally increased participant confidence and self-worth [39].

In the last ten years, there has been recognition of the significance of self-compassion—being kind and understanding to yourself—for feeling mentally and physically well. Many studies have also shown that when people are self-compassionate, they tend to have: lower stress levels [40–42], improved engagement with health-promoting behaviours [43,44] and better overall physical health and well-being [42,43] This further corroborates separate intervention studies which contended that creative arts helped adolescent participants to freely discuss and openly express their feelings regarding their illnesses [45].

Music was a notable social inclusion and unifying creative activity among participants in *Wakakosha*. Moreso, music had psychological effects as evidenced in the background music played during writing sessions which had a cathartic effect on participants as it helped them to concentrate and focus. The lyrical content of the group songs further helped participants to reflect on and verbalise their worries and aspirations in view of the self-stigmatising ideations they had prior to the intervention. It also appeared to support participants with integration of some of the deep realisations they had as a result of working with deeply rooted beliefs using IBSR. Additionally, music was educational as the song lyrics taught participants about the various challenges they encountered in view of internal stigma. This is consistent with previous studies which attested to the universality of music in self-expression and communication among adolescents and young adults with different illnesses including depression [46,47].

Singing, and more broadly music, have also many physiological benefits, such as improving breathing, posture and muscle tension, pain relief, and some evidence in sustaining a healthy immune system [48]. People also express feeling more positive after singing, more so than listening to music or talking about positive life events. Music functions cognitively to embody abstract thoughts and connect such thoughts and intuitions to the world [49]. It generates ‘musical emotions’ which help in mood regulation and expression of personal inner states and feelings especially among young people [50,51]. A systematic review of well-being outcomes for music and singing in adults concluded that despite inconsistency in how well-being was measured, there is reliable evidence for positive effects from such activities [52]. However, this review also noted that groups at greater well-being risk tended to be underrepresented in music and choirs, with participants being mostly white, female and relatively well-educated [52]. Our study addresses this gap but, since most of the songs were in English, this may have inhibited the effect.

Previous research has shown that for people with life stressors, including internal stigma, engagement in positive distractions leads to positive emotions and improved life satisfaction [28]. Some *Wakakosha* creative activities, especially colouring, helped participants by positively distracting them from the negative thoughts associated with internal stigma. Zen doodling (with roots in Zentangle) has also been noted to foster a deep meditative experience, as well as self-compassion, which has a positive effect on participants’ mental health outcomes [53,54]. This needs to be interpreted with some caution though as some scholars have dismissed the effectiveness of positive distraction in managing life stressors, arguing that it is a maladaptive stress response [55,56].

Of note, children, adolescents and young PLHIV have highlighted how the impact of activities conducted within protected spaces with individuals like them can be easily undermined by what they encounter outside the confines of these settings [12]. The socio-ecological model [57] provides a useful framework for understanding how health and well-being are influenced by multiple factors at various levels (individual, family/relationship, community, societal). In keeping with the socio-ecological model, interventions need to address multiple levels and address intersectional stigma and see how creativity can operate at, and through, all these levels.

Interestingly, *Wakakosha* creative activities were impactful to both female and male participants. Specifically, they helped younger men express their deeply seated emotions rather unconsciously within a setting where males are less able to express emotional concerns, largely due to hegemonic masculinity where they are socialised around toughness and concealing emotions [30,58,59]. Culturally sensitive ways of doing this need to be incorporated into the intervention implementation training [30], and need to be maintained/enhanced going forward.

A strength of our study is that we analysed data collected from a range of stakeholders and through various data collection methods. With specific reference to qualitative research, triangulation has been widely adopted as a means of investigating the validity of both the data and the conclusions derived from them [60–62]. In this case, there was concordance among data obtained through FGDs, IDIs and KIIs, highlighting not only the likely validity of the results but also the value of triangulation. A potential limitation is that as participants were already Zvandiri CATS with potentially lower internal stigma levels, the overall positive impact reported here may not necessarily reflect what would occur in for example, CATS clients. Also, our sample included older adolescents (18–19 years-old) who are by definition, adults and whose views and experiences may differ from those of younger adolescents. Future initiatives should evaluate impact of the intervention and embedded activities (including creativity) among CATS’ clients, younger adolescents and AYPLHIV outside of the Zvandiri program. Finally, future iterations of the programme need to contextualise the music content to improve relatability to participants for whom English is a second language.

## Conclusions

We set out to explore the integration of creative arts into IBSR, a cognitive and awareness-based internal stigma intervention among AYPLHIV in Harare, Zimbabwe. As reported by participants, creativity contributed to the internal stigma reduction process and improved overall well-being by positively influencing emotional regulation, self-acceptance, self-compassion, forgiveness, self-worth and body positivity. Findings are potentially important for health and well-being interventions in general and HIV interventions specifically, particularly as programmes shift from focusing on negative mental health indices to positive constructs.

## Supporting information

S1 Checklist Inclusivity in global research questionnaire. (DOCX)
